# Advances in Identifying the Mechanisms by Which Microorganisms Improve Barley Salt Tolerance

**DOI:** 10.3390/life14010006

**Published:** 2023-12-19

**Authors:** Zhiwei Chen, Zhenzhu Guo, Longhua Zhou, Hongwei Xu, Chenghong Liu, Xin Yan

**Affiliations:** 1 Key Laboratory of Microbiological Engineering of Agricultural Environment, Ministry of Agriculture, College of Life Sciences, Nanjing Agricultural University, Nanjing 210095, China; 2 Shanghai Key Laboratory of Agricultural Genetics and Breeding, Biotechnology Research Institute, Shanghai Academy of Agricultural Sciences, Shanghai 201106, China; gzzsk173@163.com (Z.G.); zhoulonghua@saas.sh.cn (L.Z.); xuhongwei@saas.sh.cn (H.X.); liuchenghong@saas.sh.cn (C.L.)

**Keywords:** *Hordeum vulgare* L., microbiome, salt tolerance, omics, bacteria community

## Abstract

As the global human population continues to increase, the use of saline–alkali land for food production is an important consideration for food security. In addition to breeding or cultivating salt-tolerant crop varieties, microorganisms are increasingly being evaluated for their ability to improve plant salt tolerance. Barley is one of the most important and salt-tolerant cereal crops and is a model system for investigating the roles of microorganisms in improving plant salt tolerance. However, a comprehensive review of the mechanisms by which microorganisms improve barley salt tolerance remains lacking. In this review, the mechanisms of barley salt tolerance improvement by microorganisms are summarized, along with a discussion of existing problems in current research and areas of future research directions. In particular, with the development of sequencing technology and the great reduction of prices, the use of omics can not only comprehensively evaluate the role of microorganisms but also evaluate the impact of the microbiome on plants, which will provide us with many opportunities and challenges in this research area.

## 1. Introduction

The effects of global climate change and anthropogenic activities have led to increasingly serious soil salinization issues that affect agricultural production and environments. A total of approximately 1 billion hectares of saline–alkali land has been estimated globally, with land in China accounting for approximately 10% of the global total [1]. As population growth and pressure on food security continue to increase, there is an increasing emphasis on the use of saline–alkali lands, especially for food production. However, in using saline–alkali land for crop production, the mechanism of salt tolerance must be understood so that effective measures can be taken to overcome the adverse effects of salt stress.

**Figure 1:**
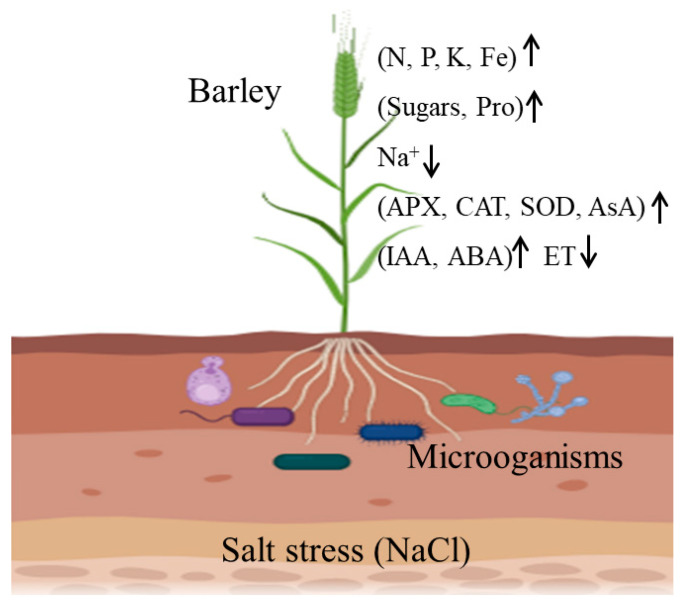
Mechanisms of microorganisms in the alleviation of salt stress in barley (created using biorender.com) (accessed on 14 November 2023).

Studies of the molecular mechanisms underlying plant salt tolerances and the breeding/cultivation of salt-resistant (SR) crop varieties have been the primary means of mitigating salinization problems. However, increasing numbers of studies have shown that microorganisms play important roles in improving the salt tolerances of plants. These benefits arise from several plant processes, including nutrient acquisition promotion, ion homeostasis maintenance, increasing osmotic substance concentrations, scavenging excess reactive oxygen species (ROS), and regulating phytohormones (Figure 1), and the mechanisms of these microorganisms in improving plant salt tolerance have also been reviewed from different perspectives [2,3,4,5]. Therefore, the use of microorganisms to improve salt tolerance in plants has been gaining traction recently, as it is considered more environmentally friendly and sustainable.

Barley is the fourth largest cereal crop globally, an important raw material in the beer industry, an important feed for animals, and a staple food in many countries [6]. At the same time, barley is also a model plant, especially for the Triticum plants, so it is also a good reference for other crops when carrying out related research. Consequently, improved salt tolerance of barley is important for mitigating salinization problems. Although a lot of research work has been carried out on the use of microorganisms in improving the salt tolerance of barley, there is still a lack of comprehensive induction or summary on improving barley salt tolerance using microorganisms, especially their mechanisms. Here, the mechanisms that can be used by microorganisms to improve the salt tolerance of barley are reviewed to provide a framework for using microorganisms to improve barley salt tolerance in addition to the comprehensive development and use of salinized soils (Table 1).

## 2. Nutrient Acquisition Promotion

Salinized soils contain abundant Na^+^ and Cl^+^ concentrations that interfere with the absorption of other nutrients by plants, resulting in nutrient deficiencies and affecting normal plant growth and development [5]. In addition, changes in pH can contribute to the loss of certain nutrients or changes in their bioavailability, further affecting their absorption by plants. Microorganisms that live on or around the surfaces of plant roots or within plant roots can enhance nutrient absorption in plants. Under salt stress, the nutrients that microorganisms help plants absorb primarily include N, P, Fe, and K. In addition, the “Ion homeostasis maintenance” section explains the competition that occurs between K^+^ and Na^+^ absorption and K^+^ absorption.

Microorganisms can help plants absorb P primarily through three pathways. First, microorganisms convert insoluble inorganic phosphates into soluble forms via acidification, chelation, ion exchange, and reduction, thereby increasing available phosphates in soils [22,23,24]. Second, microorganisms decrease the threshold of phosphorus absorption in plants by expressing the high-affinity Pi transporter, PiPT [25]. Third, microorganisms improve phosphorus uptake by plants via interactions among root endophytic fungi and phosphorus-solubilizing bacteria [26]. In support of the above assertions, inoculation of barley with the novel rhizobacterium *Siccibacter* sp. strain C2 that was isolated from the rhizosphere of other plants significantly improved barley salt tolerance, along with demonstrating exceptional phosphorus-solubilizing capacity [20].

Plants primarily absorb nitrogen (N) in the forms of nitrate (NO_3_^−^) and ammonium (NH_4_^+^), in addition to through organic compounds such as amino acids or peptides. Microorganisms primarily improve plant N uptake by (1) symbiosis via nitrogen-fixing rhizobia; (2) N-fixation by autogenous N-fixing bacteria (or non-symbiotic N-fixing bacteria); (3) by increasing the surface area of plant roots; or (4) by stimulating the expression of plant N metabolism-related enzymes that can improve N uptake and use efficiency by plants [27,28,29,30]. Non-symbiotic N-fixing bacteria are widely used to improve N uptake by plants under salt stress due to the limitations of symbiotic nodulation crops and the sensitivity of fungi to salt stress. For example, inoculation of the N-fixing bacterium *Azospirillum brasilense* into soils can significantly ameliorate the adverse effects of salt stress on barley growth and yield [9].

Iron (Fe) is also abundant in soils but not generally available to plants, especially under salt-stress conditions. Microorganisms can increase Fe availability by producing organic acids or secreting siderophores [31]. Concomitantly, siderophores can also control pathogenic bacterial growth by depriving them of Fe [32]. These processes benefit the growth and development of plants under salt stress. For example, the combined application of *Azospirillum,* mycorrhiza, and 0.9 g/L nano Fe oxide increased barley yield by approximately 15.45% under salt-stress conditions [33]. Further, *Siccibacter* sp. strain C2 can secrete siderophores, and inoculation with this rhizobacterium has been shown to significantly improve barley salt tolerance [20].

## 3. Ion Homeostasis Maintenance

Na^+^ accumulation increases its competition with K^+^ while also altering the cellular Na/K ratio and, in turn, disrupting enzyme activity, protein synthesis, turgor maintenance, photosynthesis, and stomatal motility [34]. A high Na/K ratio in plants indicates high salt stress, such that plants must maintain low levels of Na^+^ to resist their harmful effects on plants. Microorganisms can improve plant salt tolerance by limiting the uptake of Na^+^ in roots while increasing K^+^ uptake.

During salt stress, inoculation with arbuscular mycorrhizal fungi (AMF) significantly increases K^+^ uptake while decreasing Na^+^ uptake, thereby decreasing the Na/K ratio and improving the salt tolerance of plants [35,36]. Further, K^+^ uptake increased with increasing salinity due to the mycorrhiza [35]. The high-affinity K^+^ transporter HKT1 controls Na^+^ input in plant roots, while HKT1 overexpression in plants does not improve its salt tolerance, and inoculation with *Bacillus subtilis* GB03 can downregulate and upregulate *HKT1* expression in roots and shoots, respectively, thereby reducing Na^+^ concentrations in the whole plant and improving plant salt tolerance [37]. These results highlight the complex roles of microorganisms in regulating salt tolerance in plants.

Colonization of *Piriformospora indica* in plant roots under salt stress resulted in a lower Na/K ratio, which might be due to the upregulation of *HKT1* and the genes *KAT1* and *KAT2* that encode potassium channel proteins [38]. Inoculation of an endophytic fungus (*Neotyphodium* spp.) reduced Na^+^ concentrations in plant roots while increasing K^+^ concentrations in shoots, thereby increasing the K/Na ratio of plants and alleviating salt-stress damage to plants [39]. A previous study aimed at improving plant salt tolerance by PGPB observed that maize seedlings inoculated with *Bacillus amyloliquefaciens* SQR9 could reduce plant Na^+^ content and improve their salt tolerance under salt-stress conditions. However, no effect on K^+^ concentrations was observed, and thus, the altered Na/K ratio was primarily due to a reduction in Na^+^ concentrations [40]. The formation of biofilms under various stress conditions is an important strategy for improving the survival of bacteria in plant rhizospheres. Microorganisms can secrete abundant extracellular polymers (primarily extracellular polysaccharides or EPS) that lead to biofilm formation on the surfaces of plant roots after the inoculation of bacteria onto plants, thereby hindering Na^+^ entry. For example, Ashraf et al. [41] inoculated EPS-producing bacteria in wheat, observing that it limited the uptake of Na^+^ by roots, thereby improving plant salt tolerance.

Several studies have evaluated these effects in barley. For example, the K/Na ratio in barley leaves increased due to decreasing accumulation of Na^+^ during salt stress after inoculation with a root endophytic fungus (*Piriformospora indica*), while barley salt tolerance was also improved according to their biomass [10,12]. Sepehri et al. [19] observed similar results after inoculating the root endophytic fungus *Serendipita indica* into barley. However, no effect on the concentrations of Na^+^ and K^+^ was observed in leaves or leaf sheaths after inoculation with plant rhizosphere bacteria, although root Na^+^ uptake was reduced [13]. An additional study indicated that inoculation of PGPB reduced Na^+^ concentrations in both the roots and shoots of barley during salt stress while increasing the water potential of leaves, thereby alleviating salt-stress damage to plants [16]. Moreover, inoculation of microorganisms with the ability to produce biofilms also improved barley salt tolerance, although the precise effects of biofilms on Na^+^ content were not measured [14,15].

## 4. Increasing Osmotic Substance Concentrations

Under salt stress, plants typically synthesize many soluble small organic molecule compounds as osmolytes, like proline, glycine betaine, sugars, organic acids, polyamines, and amino acids. These osmolytes are also involved in scavenging ROS, the maintenance of membrane integrity, and the stabilization of enzymes, thereby also acting as osmoprotective compounds.

Many microorganisms can produce proline. The *proBA* genes of *Bacillus subtilis* strain 93151 have been overexpressed in *Arabidopsis thaliana* by using the 35S promoter, leading to significantly higher proline content in transgenic plants than in control plants, with salt and drought stress tolerance both being enhanced in the former [42]. Moreover, inoculation of the rhizosphere bacteria *Staphylococcus haemolyticus* strain ST-9 and *Bacillus subtilis* strain RH-4 isolated from the rhizosphere of *Heleochloa schocnoides* significantly improved the proline content and salt tolerance of chickpea plants [43]. However, some studies have suggested that proline content in rice increases under salt stress, while inoculation with plant growth-promoting bacteria (PGPB) or their mixtures with endophytic bacteria (e.g., *Pseudomonas pseudoalcaligenes* and *Bacillus pumilus*) reduces proline content in rice but enhances rice salt tolerance [44]. Consequently, the role of microorganisms in proline accumulation within salt-stressed plants remains controversial, including for barley plants. Inoculation of *Azospirillum brasilense* into barley improved its salt tolerance, with decreased proline accumulation implicated as one of the underlying causes [9], consistent with the results of Badawy et al. [18]. Nevertheless, proline production was also significantly higher in plants during salt-stress alleviation and after inoculation of the bacterial strains *Bacillus mojavensis* S1, *B. pumilus* S2, and *Pseudomonas fluorescens* S3 into soils [16].

Soluble sugars (e.g., glucose, sucrose, dextrin, and maltose) also act as osmoprotective compounds by stabilizing cell membranes and protoplasts while also protecting enzymes from high intracellular concentrations of inorganic ions [45]. Under high salinity conditions, plant sucrose is broken down to meet glucose requirements [46]. Inoculation of cucumber seedling leaves with the siderophore-producing bacterial strain *Trichoderma asperellum* Q1 significantly increased the soluble sugar concentrations in both control and salt-stress conditions [47]. Likewise, barley inoculated with *Piriformospora indica* under variable salt stresses exhibited enhanced salt tolerance and increased soluble sugar concentrations [12]. Further, seawater stress led to decreased soluble sugar concentrations in barley, while inoculation with *Aspergillus ochraceus* effectively alleviated the adverse effects of salt stress on barley and also increased soluble sugar concentrations in barley leaves under different treatments, thereby suggesting a role of soluble sugars in barley salt tolerance [18].

## 5. Scavenging Excess ROS

Salt stress induces ROS production, including production of superoxide radicals (O_2_^−^), hydroxyl radicals (OH^−^), and hydrogen peroxide (H_2_O_2_), resulting in oxidative damage to lipids, proteins, and DNA [48]. Two approaches are primarily used by plants to cope with the adverse effects of excessive ROS, namely enzymatic and non-enzymatic antioxidant systems [2]. Enzymatic systems primarily include ascorbate peroxidase (APX), catalase (CAT), superoxide dismutase (SOD), glutathione reductase (GR), dehydroascorbate reductase (DHAR), and monodehydroascorbate reductase (MDAR). In addition, non-enzymatic antioxidant systems primarily include ascorbic acid (AsA), glutathione (GSH), carotenoids, and several types of osmolytes. Microorganisms can activate antioxidant defense mechanisms by upregulating the activities of antioxidant enzymes or antioxidant concentrations in plants to resist damage caused by excessive ROS [5].

Inoculation with *Trichoderma asperelloides* T203 affects the expression of genes associated with osmotic protection and oxidative stress in the roots of two kinds of plants, with genes encoding monodehydroascorbate reductase (MDAR) significantly upregulated in the plants and increased ascorbic acid concentrations also observed [49]. Further, inoculation with *Piriformospora indica* increased the concentration of non-enzymatic antioxidants like carotenoids and proline in rice enduring salt stress, thereby improving its salt tolerance [50]. Inoculation with *Trichoderma longibrachiatum* T6 also increased the expression of genes encoding the antioxidant enzymes superoxide dismutase (SOD), peroxidase (POD), and catalase (CAT) in wheat seedlings undergoing salt stress, thereby improving their salt tolerance [51]. Inoculation with the SR PGPB *Dietzia natronolimnaea* STR1 enhanced the gene expression of multiple antioxidant enzymes like APX, MnSOD, CAT, POD, GPX, and GR, thereby increasing proline content and protecting wheat from the adverse effects of salt stress [52]. Moreover, inoculation with *Azospirillum lipoferum* FK1 increased the levels of enzymatic and non-enzymatic antioxidants in chickpea seedling leaves, thereby improving their salt tolerance [53]. In addition, the activities of superoxide dismutase (SOD), peroxidase (POD), and catalase (CAT) increased in the roots of wheat during salt stress after inoculation with the PGPB *Nesterenkonia rhizosphaerae* wp-8, while the proline contents of leaves also significantly increased and malondialdehyde concentrations significantly decreased. These results indicate that the bacterium could alleviate the adverse effects of salt stress and promote seedling growth and root development by regulating antioxidant systems [54]. Inoculation with the PGPB *Pseudomonas pseudoalcaligenes* and *Bacillus pumilus* helps to reduce lipid peroxidation and superoxide dismutase activities in the salt-sensitive (SS) rice variety GJ17, thereby improving its adaptation to salt stress [55]. Most studies have reported that inoculation with microorganisms might be associated with increased antioxidant levels, although direct evidence is lacking to confirm the relationship between any antioxidant enzymes and plant salt tolerance.

In barley, many studies have investigated the relationship between improving salt tolerance by inoculation of microorganisms and antioxidants. Barley salt tolerance was improved after inoculation with *Piriformospora indica* and might be related to increased antioxidant capacity due to activation of the glutathione–ascorbic acid cycle [7,8]. Inoculation with the PGPB *Pseudomonas fluorescens* SBW25 and *Pseudomonas putida* KT2440 to investigate improved salt tolerance in barley revealed that both led to the upregulation of genes encoding GR and that strain SBW25 could also enhance the expression of genes encoding CAT2. Consequently, these bacteria could enhance plant salt tolerance by regulating the expression of genes encoding antioxidant enzymes [17]. Further, inoculation with *Siccibacter* C2 might alleviate oxidative stress by reducing hydrogen peroxide and malondialdehyde concentrations resulting from salt stress, thereby improving barley salt tolerance [20]. Further, inoculation with *P. macrospinosa*, *N. goegapense*, and *N. chichastianum* all increased the activities of the antioxidant enzymes SOD, CAT, and POX in barley [21]. However, the improvement of barley salt tolerance after inoculation with *Azospirillum brasilense* could be related to decreased antioxidant enzyme activity that was more effective in the SS rice variety [9]. In addition to the above, inoculation with *Aspergillus ochraceus* mitigated the harmful effects of seawater on the growth and physiologic status of barley plants while also leading to increases in proline, malondialdehyde, and hydrogen peroxide concentrations, in addition to antioxidant enzyme activities [37]. The results above demonstrate that varying relationships exist between microbial-induced changes in antioxidants and barley salt tolerances.

## 6. Regulating Phytohormones

Phytohormones play important roles in regulating the growth and development of plants while also helping alleviate abiotic stresses. Phytohormones are commonly considered plant growth regulators that primarily include abscisic acid (ABA), gibberellin (GA), ethylene (ET), cytokinins, and auxin (especially indoleacetic acid or IAA). Some microorganisms can produce or regulate phytohormones that can help plants tolerate or avoid salt stress. For example, inoculation with *Pseudomonas putida* Rs-198 increased the germination rate and seedling growth of cotton during salt stress while concomitantly leading to increased IAA concentrations and decreased ABA concentrations in plants [56]. Inoculation of cucumbers with three plant growth-promoting bacteria, namely *Burkholdera cepacia* SE4, *Promicromonospora* sp. SE188, and *Acinetobacter calcoaceticus* SE370 strain, to evaluate their effects during salt stress suggested that they might be associated with the downregulation of ABA and the upregulation of SA and GA4 in plants [57]. Further, *Trichoderma asperellum* Q1 can produce IAA, GA, and ABA, and inoculation of plants with this strain increased the concentrations of these three phytohormones and alleviated salt damage to cucumber seedlings [58]. Transcriptome analysis also revealed that inoculation with *Bacillus amyloliquefaciens* FZB42 induced transcription of ET and jasmonic acid (JA)-related genes in *Arabidopsis thaliana* during salt stress, while the inoculation of *Arabidopsis*-related mutations further confirmed that strain FZB42 might induce salt tolerance in plants by activating plant ET and JA signaling rather than through an ABA-dependent pathway [59]. However, inoculation with the PGPB *B. amyloliquefaciens* RWL-1 strain that can produce ABA in rice plants experiencing increasing salt concentration stress led to decreased ability to produce ABA (and decreased concentrations in plants) but significantly improved salt tolerance, with SA concentrations also increasing [60]. Thus, plants often exhibit variable concentrations of phytohormones after inoculation with microorganisms, and inoculation with microorganisms that produce certain phytohormones does not necessarily lead to their increased concentrations in plants. Consequently, the specific relationships among microbial hormone production, their regulation of plant endogenous hormones, and plant salt tolerance require further study.

PGPB that produce 1-aminocyclopropane-1-carboxylate (ACC) deaminase are thought to reduce ethylene levels and improve plant growth under salt stress because the salt tolerance of barley was improved after inoculation with some ACC deaminase-producing microorganisms [11]. In one study, barley seedlings inoculated with *Hartmannibacter diazotrophicus* E19^T^ were exposed to 200 mM and 400 mM NaCl for 2 h, revealing reduced ethylene release and improved salt tolerance, indicating that this might be due to ACC deaminases produced by microorganisms [45]. An investigation of the molecular mechanism of *Pseudomonas fluorescens* SBW25 and *Pseudomonas putida* T2440 in improving salt tolerance of barley indicated the presence of greater downregulated genes associated with ABA biosynthesis and regulation but more upregulated genes related to JA, ethylene, and SA biosynthesis [17]. Further, inoculation of the PGPB *Bacillus mojavensis* S1 and *Pseudomonas fluorescens* S3 alleviated damage from salt stress to barley and induced plants to produce large amounts of IAA [38].

## 7. Challenges and Perspectives

Increasing emphasis on environmental protection and agricultural sustainability has led to an increased interest in addressing the adverse effects of salt stress on plants in a cost-effective manner. Given that microorganisms can improve the tolerance of crops to salt stress, understanding these mechanisms will help lead to improved crop growth and development, in addition to increased crop yields. Although many studies have identified the mechanisms by which microorganisms improve plant salt tolerance, most have been limited to the inoculation of individual microorganisms. Yet, hundreds of millions of microorganisms are present in soils within rich and diverse communities. Nevertheless, it is unclear whether inoculation by a single isolate would be viable in a natural field environment and whether those populations can survive or exist in large abundances. Overall, practical application cases in fields are still lacking to assess these knowledge gaps. Microbial-mediated salt tolerance in plants is very complex, and the investigation of several indicators or traits is not enough to comprehensively evaluate the specific roles of microorganisms, leading to the generation of partial or contradictory conclusions. In addition, most of the microorganisms that improve barley salt tolerance are not derived from barley or their growing environments, and whether the use of exogenous microorganisms is the most suitable method for barley remains to be investigated.

**Figure 2:**
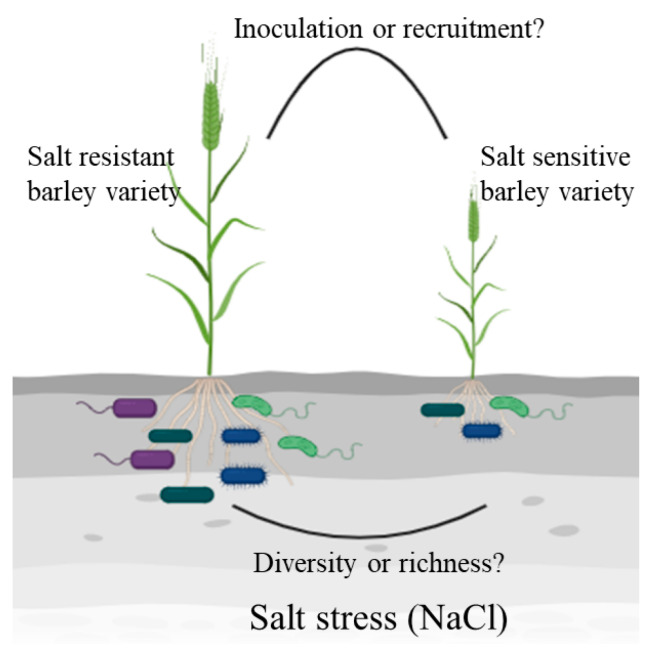
Plant–microbe interaction under salt stress in barley (created using biorender.com) (accessed on 17 November 2023).

The development of sequencing technologies and the concomitant greatly reduced costs have allowed the roles of microbial communities in plant salt tolerance to be clarified at the level of the microbiome. Zheng et al. [61] compared differences in rhizobia microbiomes between two wild halophytic legumes and a soybean cultivar using PacBio sequencing of 16S rRNA genes, revealing that the salt tolerances of wild legumes might be related to the richness of rhizobia in nodules. When comparing the composition and changes of rhizospheric and endophytic bacteria in SS and SR plants, specific bacteria were recruited that enabled each plant to grow, although SS and SR recruited different bacteria when subjected to salt stress [62]. Further, this bacterial recruitment primarily occurred in the rhizosphere rather than within the root, leading to the conclusion that the microbial communities, rather than the individual populations, exhibited persistent resistance to salt stress. Wang et al. [63] proposed a new model of “amplification-selection” for bacterial community assembly by applying an absolute quantitative method, subsequently suggesting that rhizospheric bacteria were the most abundant in soil environments and thus may play more important roles. These studies provide better insights for further research into the roles of microorganisms in improving barley salt tolerance. A recent investigation that inoculated *Trichoderma harzianum* T-22 into SR and SS barley varieties undergoing salt stress was conducted, with GC-MS and LC-MC used to compare the effects of the bacterium on barley metabolic changes, resulting in a more comprehensive evaluation of the microbial effects on barley physiology [64]. The use of other multi-omics methods, including transcriptomics, proteomics, ionomics, and others, will help develop a more comprehensive understanding of microbial effects on plants. In addition, rice encoding the *NRT1.1B* gene can recruit specific rhizosphere microbial communities that can improve the N use efficiency of rice, with the role of rhizosphere-specific enriched microbial communities in N uptake confirmed using synthetic bacterial communities [65]. Such experiments comparing the microbiomes of SR and SS barley varieties provide new directions for identifying microbial communities that can improve barley salt tolerance, and further research is needed to determine whether it is more important to improve salt tolerance by inoculating beneficial microorganisms directly or by increasing the recruitment of beneficial microorganisms by the plants themselves (Figure 2).

## 8. Conclusions

This review summarizes the main mechanisms of microorganisms in improving the salt tolerance of barley and will provide some references for those who are starting research in these areas. Due to the concentration and timing of salt treatment, the use of different barley varieties, and the fact that most of them are based on individual microorganisms, as well as some phenomena that seem contradictory in specific cases, the mechanism of microorganisms in barley salt tolerance remains to be further investigated. With the development and application of omics, it is expected to be used to evaluate the role of microorganisms in improving the salt tolerance of barley and the interaction patterns between plants and microorganisms from a more comprehensive perspective.

## Figures and Tables

**Table 1:** Summary of beneficial effects of microorganisms on barley under salt stress.

Barley Variety	Microorganism Species	Effect or Mechanism	Reference
Ingrid, Annabell	*Piriformospora indica*	Increasing ascorbate content and DHAR activity	[7]
Ingrid, California Mariout	*Piriformospora indica*	Increasing plant growth and attenuating NaCl-induced lipid peroxidation, metabolic heat efflux and fatty acid desaturation in leaves of the salt-sensitive barley cultivar Ingrid, elevating the amount of ascorbic acid and increasing the activities of antioxidant enzymes in barley roots	[8]
Giza 123 and 2000	*Azospirillum brasilense* (NO40)	Ameliorating the adverse effect of salinity on growth and yield, increasing pigment contents, reducing accumulation of the osmoregulator proline and activities of antioxidant enzymes	[9]
Pallas	*Piriformospora indica*	Increasing the foliar K^+^/Na^+^ ratio and Ca^2+^ content	[10]
Ranger	*Pseudomonas* sp. UW3 and UW4	Increasing dry masses	[11]
Pallas	*Piriformospora indica*	Increasing K^+^/Na^+^ and Ca^2+^/Na^+^ ratios and the sugars and free amino acid contents	[12]
Propino	*Hartmannibacter diazotrophicus* E19^T^	Reducing ethylene emission	[13]
Giza 123 and 2000	HM6 (B6) (*Bacillus amyloliquifaciens*)	Forming biofilms	[14]
Giza123	*Bacillus amyloliquefaciens* (HM6) mutaions	Inhibiting POX and CAT activities while increasing AsA content	[15]
Rihane	*Bacillus mojavensis* (S1), *Bacillus pumilus* (S2), *Pseudomonas fluorescens* (S3)	Increasing shoot and root dry weights and proline concentrations (S1 and S3) while reducing leaf water potential and shoot and root Na^+^ concentrations	[16]
	*Pseudomonas fluorescens* SBW25 and *Pseudomonas putida* KT2440	Enhancing root fresh and dry weights, chlorophyll content, and relative water content	[17]
Giza 111	*Aspergillus ochraceus* MT089958	Mitigating the harmful effects of seawater on the growth and physiology of barley plants, including reducing the free proline	[18]
Pallas	*Serendipita indica* (*Piriformospora indica*)	Decreasing shoot Na^+^ content	[19]
Rihane	*Siccibacter* sp. Strain C2	Increased proline and total soluble sugar contents and chlorophyll while decreasing hydrogen peroxide and malondialdehyde contents	[20]
Reyhan 03	*Periconia macrospinosa, Neocamarosporium goegapense, and N. chichastianum*	Enhancing biomass, shoot length, chlorophyll concentration, proline content, and activities of catalase, peroxidase, and superoxide dismutase	[21]

## Data Availability

Not applicable.
